# The *Bacillus Subtilis* K-State Promotes Stationary-Phase Mutagenesis via Oxidative Damage

**DOI:** 10.3390/genes11020190

**Published:** 2020-02-11

**Authors:** Holly A. Martin, Amanda A. Kidman, Jillian Socea, Carmen Vallin, Mario Pedraza-Reyes, Eduardo A. Robleto

**Affiliations:** 1 University of Nevada, Las Vegas, 4505 S Maryland Pkwy, Las Vegas, NV 89154, USA; mart1015@unlv.nevada.edu (H.A.M.); akidman@unlv.nevada.edu (A.A.K.); soceaj@unlv.nevada.edu (J.S.); vallincarmen@gmail.com (C.V.); 2 Department of Biology, Division of Natural and Exact Sciences, University of Guanajuato, P.O. Box 187, Guanajuato Gto. 36050, Mexico; mario_pedraza@hotmail.com

**Keywords:** competence, K-state, stationary-phase mutagenesis, ComEA, ComK

## Abstract

Bacterial cells develop mutations in the absence of cellular division through a process known as stationary-phase or stress-induced mutagenesis. This phenomenon has been studied in a few bacterial models, including *Escherichia coli* and *Bacillus subtilis*; however, the underlying mechanisms between these systems differ. For instance, RecA is not required for stationary-phase mutagenesis in *B. subtilis* like it is in *E. coli*. In *B. subtilis*, RecA is essential to the process of genetic transformation in the subpopulation of cells that become naturally competent in conditions of stress. Interestingly, the transcriptional regulator ComK, which controls the development of competence, does influence the accumulation of mutations in stationary phase in *B. subtilis*. Since recombination is not involved in this process even though ComK is, we investigated if the development of a subpopulation (K-cells) could be involved in stationary-phase mutagenesis. Using genetic knockout strains and a point-mutation reversion system, we investigated the effects of ComK, ComEA (a protein involved in DNA transport during transformation), and oxidative damage on stationary-phase mutagenesis. We found that stationary-phase revertants were more likely to have undergone the development of competence than the background of non-revertant cells, mutations accumulated independently of DNA uptake, and the presence of exogenous oxidants potentiated mutagenesis in K-cells. Therefore, the development of the K-state creates conditions favorable to an increase in the genetic diversity of the population not only through exogenous DNA uptake but also through stationary-phase mutagenesis.

## 1. Introduction

For over 60 years, genetic experiments have shown that bacterial cells can develop mutations in the absence of growth; this is known as stationary-phase mutagenesis or stress-induced mutagenesis [1,2,3]. This type of mutagenic processes is independent of growth, and is observed in bacteria and eukaryotes. Therefore, they expand our view of the evolutionary process and can explain the formation of mutations that confer antibiotic resistance and cancers [1]. However, the cellular mechanisms governing this ability to undergo mutagenesis during stress remain unclear, particularly in bacterial models other than *Escherichia coli*. One interesting question in the stationary-phase mutagenesis field is how a bacterial population manages increases in genetic diversity without increasing the chance for genetic load. In this regard, research has examined two aspects: (1) whether all the cells in a population are subject to mutagenesis, and (2) whether all the genome is subject to mutagenesis. In *E. coli*, stressed cells have evolved mechanisms to license the increase of error-prone repair at genetic regions surrounding DNA double-stranded breaks [4], which occur at a 10^−3^ frequency spontaneously [5]. Repair of double-stranded breaks, processed by recombination, can generate point mutations produced via error-prone synthesis or genetic amplifications that confer fitness to cells experiencing limited replication [6,7,8]. Interestingly, genetic regions undergoing transcription in stressed cells can precipitate the formation of double-stranded breaks via the formation of R-loops [9]. Also, factors affecting transcription termination influence stress-induced mutagenesis [10]. Therefore, the observations above support a model in which stressed *E. coli* manage increases in genetic diversity by activating mechanisms that operate on a subpopulation of cells and transcribed DNA undergoing repair of double-stranded breaks [1].

In the context of increasing genetic diversity in times of nutritional stress, *B. subtilis* halts replication and differentiates subpopulations [11,12]. One subpopulation of cells become naturally competent, a strategy to acquire new genes from the environment by the process of recombination [13]. Interestingly, *Bacillus subtilis* also expresses stationary-phase mutagenesis. In these processes, the transcription repair coupling factor Mfd plays a central role, interacting with components of the nucleotide excision repair (NER) or base excision repair (BER) pathways and error-prone polymerases to produce mutations in stationary-phase cells [14]. This type of mutation occurs under endogenous levels of DNA damage or when cells are exposed to oxidants that inflict DNA lesions. These observations support a mutagenic model in which actively transcribed genes accumulate more mutations than those that are repressed through error-prone repair. However, whether other post-exponential processes, including the development of a competent subpopulation, contributes to stationary-phase mutagenesis remains an open question.

Sung and Yasbin contributed two interesting observations to our understanding of stationary-phase mutagenesis [2]. RecA, and therefore the process of genetic recombination, is not required for this process. Also, inactivation of ComK, a transcriptional regulator that controls the late steps in the development of the competence subpopulation, decreased stationary-phase reversions to methionine prototrophy. Moreover, the inactivation of ComA, which controls the early steps in competence development, did not affect Met^+^ reversions [2]. These observations suggest that the genetic changes producing Met^+^ reversions in stationary-phase *B. subtilis* are promoted by the late steps that differentiate cells into competence in the absence of genetic recombination.

Characterization of the formation of the competent subpopulation has redefined this cell differentiation pathway as a mechanism to cope with stress that goes beyond promoting genetic recombination [15]. Transcriptomics studying the K-state (the competent state) show that recombination genes are only a subset of genes activated during competence. K-cells also express factors that detoxify cells, facilitate uptake and use of nutrients, and repair DNA lesions [15]. Given these observations, we examined the idea that the development of the K-state leads to conditions that predispose cells to accumulate stationary-phase mutations even in the absence of DNA uptake that provides the substrate for genetic recombination.

Our experiments showed that cells that undergo genetic transformation are more likely to become stationary-phase mutants and that oxidative damage to DNA is a precursor to the formation of mutations. Furthermore, stationary-phase Met^+^ mutagenesis occurs in K-cells that are deficient in the uptake of DNA, but at slightly lower levels than in cells containing a functional uptake apparatus (ComEA^+^ cells). To better study this phenomenon, we used a ComK-inducible system to turn all the cells in the culture into the K-state and measure mutagenesis. We found that ComEA^−^ cells produced fewer stationary-phase mutants than their ComEA^+^ counterparts but tolerated oxidant exposure better than ComEA^+^ cells. Furthermore, K-cells showed better survival to oxidative stress than non-K-cells, which indicated that this differentiation state activates mechanisms that repair oxidative damage. We followed these observations with microscopy assays of K-cells differing in ComEA and measured fluorescence of an indicator dye as a proxy for oxidative damage. These assays showed that, under oxidative stress, ComEA^−^ cells were less damaged than ComEA^+^ cells. These results support the concept that developing into the K-state, which includes installing a functional DNA uptake system in the cell surface, predisposes *B. subtilis* to oxidative damage. K-cells activate mechanisms to survive oxidative damage and increase mutagenesis in cells under non-lethal selection pressure. Therefore, *B. subtilis* K-cells increase genetic diversity via recombination-dependent and recombination-independent pathways. 

## 2. Materials and Methods 

### 2.1. Bacterial Strains and Growth Conditions

*B. subtilis* strains used in this study are all derivatives of the strain YB955 and are described in Table 1. YB955 is a prophage-cured 168 strain that contains the following auxotrophic genes: *hisC952*, *metB5*, and *leuC427* [2]. *B. subtilis* strains were all maintained on tryptose blood agar base medium (TBAB; Difco Laboratories, Sparks, MD, USA) or grown in liquid cultures in Penassay broth (PAB) (antibiotic A3 medium; Difco Laboratories, Sparks, MD, USA) with antibiotics, such as 5 μg/mL of neomycin (Nm), 100 μg/mL of spectinomycin (Sp), 5 μg/mL chloramphenicol (Cm), or 10 μg/mL of tetracycline (Tc), as needed. *Escherichia coli* strains were maintained and grown on Luria–Bertani (LB) with antibiotics as needed.

The strain containing a defective *comEA* gene (JC101) was constructed by cloning a neomycin cassette within the *comEA* gene. Two sets of primers (see Table 2) were designed to amplify regions of *comEA* from YB955. The primers were flanked with restriction sites compatible with plasmid pBest502 [19]. The fragments were digested with the appropriate restriction enzyme sites and ligated to pBest502. The ligation product was transformed into *E. coli* Mon1 competent cells according to the manufacturer’s recommendations (Monserate, San Diego, CA, USA). Transformants were selected on ampicillin and screened by restriction enzyme analysis. This plasmid was transformed into YB955, as previously described [20]. Briefly, *B. subtilis* YB955 was grown to T_90_, ninety minutes after the cessation of growth (stationary phase), in GM1 broth (0.5% dextrose, 0.1% yeast extract, 0.2% casein hydrolysate, essential amino acids 50 μg ml^−1^, 1X Spizizen salt solution [21] and then diluted 10-fold into GM2 broth (GM1 broth plus 50 μM CaCl_2_, 250 μM MgCl_2_). After one hour of incubation at 37 °C with aeration, plasmid DNA (100 ng) was added. The culture was incubated for another hour, followed by the addition of 100 μL of 10% yeast extract. After an additional hour of incubation, the cells were plated on TBAB containing 5 μg/mL neomycin to select for the *comEA::neo^R^* allele. Then *comEA^-^* transformants were screened by PCR.

To construct the *comK-gfp* IPTG-inducible strain, genomic DNA was isolated from *B. subtilis* BD4010^21^ using the Wizard® Genomic DNA Purification Kit (Promega, Madison, WI, USA). Isolated genomic DNA was then transformed into YB955 using the competence procedures for *B. subtilis* described above [20]. Cells were plated on TBAB containing 100 μg/mL spectinomycin to select for the *amyE::pHS-comK* allele and 5 μg/mL chloramphenicol to select for the *comK-gfp* allele. Transformants were verified by flow cytometry using IPTG and comparing the fluorescence intensity of induced versus uninduced cells in relation to parental cells (YB955).

To construct the *gfp-comK* IPTG-inducible *comEA* knockout strain, genomic DNA from JC101 was extracted using Wizard® Genomic DNA Purification Kit (Promega, Madison, WI, USA). The genomic DNA was then transformed into HAM501 using the competence protocol described above. Cells were plated on TBAB containing 100 μg/mL spectinomycin, 5 μg/mL chloramphenicol, and 5 μg/mL neomycin. Transformants were verified using the transformation assay described above and transformed using the plasmid pDG1664 [18] containing erythromycin resistance. The inability to be transformed was used to confirm the desired genotype.

### 2.2. Stationary-Phase Mutagenesis Assay

As previously described^2^, one colony of each strain was used to inoculate two mL of PAB. This was grown overnight in a shaking incubator at 37 °C with aeration (250 rpm). The next day, 1 mL of the overnight culture was transferred into 20 mL of PAB with 20 µL of 1000× Ho-Le trace elements into a 250-mL Erlenmeyer flask. Growth was monitored with a Genesys 10S UV–vis (Thermo Scientific, Waltham, MA, USA) until T_90_. Cells were then harvested by centrifugation at room temperature and resuspended in 1× Spizizen minimal salts. Cells were then plated in quintuplet (100 µL per plate) on 1× Spizizen minimal medium (SMM; 1× Spizizen salts supplemented with 0.5% glucose, 50 μg/mL of both isoleucine and glutamic acid and either 50 μg/mL or 200 ng/mL of methionine, histidine, and leucine. The lower concentration of amino acid was used to select for revertants. These plates were incubated for nine days with revertants scored every day. To determine the initial number of cells plated, the cell suspension was serial diluted by 6 log_10_ units and plated onto SMM containing 50 μg/mL of each required amino acids. The experiments were repeated at least three times.

To determine the viability of the non-revertant background cells, agar plugs were removed from each minimal plate using Pasteur pipettes every other day. These plugs were taken from areas of the plate that contained no visible colonies. The plugs were resuspended in 0.5 mL of 1× SMS solution, then serially diluted by 4 log units, and plated on SMM containing all the essential amino acids. These plates were incubated at 37 °C for two days before being colonies were counted.

### 2.3. Oxidative-Stress Induced Stationary-Phase Mutagenesis Assay

To determine the effects of oxidative stress on *B. subtilis*, cells were grown as described in the stationary-phase mutagenesis assay with the following differences. When using the ComK-inducible strains, cells were propagated to an OD_600_ of 0.75; at this point, the cultures were divided in half and one portion was induced with 1 mM IPTG. When both cultures reached T_90_ (90 minutes after the cessation of growth), they were treated with 0 or 1.5 mM *tert*-butyl hydroperoxide (*t*-BHP; Luperox(R) Sigma-Aldrich, St. Louis, MO, USA) for two hours, washed with 1X SMS twice, then plated, and assayed for stationary-phase mutagenesis.

### 2.4. Transformation Stationary-Phase Mutagenesis Assay 

Strains were grown overnight in GM1 with Ho-Le trace elements in a 250 mL Erlenmeyer flask on a bench top at room temperature for approximately 16 hours. The next day, the culture was incubated in a shaker at 37 °C with aeration (250 rpm). Growth was monitored with a Genesys 10S UV–vis (Thermo Scientific, Waltham, MA, USA) until T_90_. The culture was then diluted 10-fold into GM2 and incubated for one hour as previously described. Finally, the culture was split into two different batches. Then, 150 ng of the transforming DNA (pDR111; a gift from Dr. David Rudner) was added to one subset of the culture, while nothing was added to the other subset of the culture, which served as the no-DNA control. In addition, a broth-only control was used.

Cells were incubated for an additional hour, followed by the addition of 10% yeast extract to all three subsets. Cells were incubated one final hour and subsequently harvested. Cells were harvested by centrifugation at room temperature and resuspended in 1× SMS. Cells from the DNA treatment and no DNA cultures were plated in quintuplet (100 μL per plate) on both SMM and TBAB with 100 ng/mL of spectinomycin, which assessed transformation efficiency and spontaneous spectinomycin reversion. The SMM plates were incubated for 9 days with revertants scored every day. To determine the initial number of cells plated, the beginning cell suspension was serially diluted by 6 log_10_ units and plated onto medium containing all the required amino acids. The experiments were repeated at least three times. The viability of the cells of the non-revertant background cells was assessed as described above.

To determine if prototrophic revertants were also transformants, colonies were screened for growth on TBAB with 100 ng/mL of spectinomycin. As a control, non-revertant colonies (colonies that arose from plugs that were grown onto minimal plates containing all essential amino acids) were subject to the same screening.

### 2.5. Flow Cytometry

*B. subtilis* strains, HAM501 and AAK502, were grown to mid-exponential phase and T_90_. A batch of cells were divided into two sets at a late exponential phase (0.7–0.8 OD_600_) and IPTG was added to one set. Then, both sets (with and without IPTG) were grown until T_90_ (stationary phase). A 1-mL aliquot from each condition was removed at a late exponential phase, as well as the stationary phase, and combined with 50 μL of 37% formaldehyde. This mixture was centrifuged for 10 min at 300× *g* (2100 rpm) in a microcentrifuge tube. The supernatant was decanted, and the pellet was resuspended in 1 mL of phosphate-buffered saline (PBS). Samples were covered in foil until they were run through the BD FACSCalibur flow cytometer.

### 2.6. Fluorescence Microscopy

One colony of each strain was used to inoculate 2 mL of PAB. This culture was grown overnight in a shaking incubator at 37 °C with aeration (250 rpm). One milliliter of the overnight culture was transferred into 20 mL of PAB with 20 µL of 1000× Ho-Le trace elements into 250 mL Erlenmeyer flask. Growth was monitored with a Genesys 10S UV–vis (Thermo Scientific, Waltham, MA, USA) until OD_600_ of approximately 0.75. Then cultures were divided into halves, and one half was induced with 1 mM IPTG. We monitored the growth of the cultures until the transition from exponential to stationary phase was identified (typically around an OD_600_ of 1.5). At T_50_ (50 minutes after the cessation of growth), each condition was pre-labeled by adding 5 μM peroxy-orange 1 (PO1, Tocris, Bristol, UK). PO1 measures the concentration of H_2_O_2_ since PO1 only becomes fluorescent after oxidation by H_2_O_2_ which removes a protective boronate side group [22]. Therefore, we can assay the ROS levels of a cell by pre-loading cells with PO1 and subsequently exposing them to an oxidant like *t*-BHP. This technique has been used to measure ROS in different cell types [22,23]. Cultures were returned to the incubator and grown to T_90_. At this point, each condition was divided and exposed to either 0 or 5 mM of *t*-BHP for 2 hours. Cells were then harvested by centrifugation, washed twice with 1X SMS, and serially diluted. 

To determine percent survival, we plated 0.1 mL of serial 10-fold dilutions onto TBAB, incubated the plates for 24 hours at 37 °C, and scored for colonies to determine survival. Percent survival was determined by dividing the number of CFUs from the exposed conditions by the number of CFUs from the 0 mM condition. We repeated this procedure at least three times, with three replicates each time.

To visualize the amount of ROS in the cell, 0.8 μL of a 10^−1^ diluted culture was immobilized in a 1% agarose pad on a clean glass slide before imaging. Live cell imaging was conducted using transmitted light and fluorescence microscopy (Zeiss Axio Upright Imager M2, Oberkochen, Germany)equipped with oil immersion (Plan-Apochromat 63×/1.40 Oil Ph3 M27). Fluorescence excitation was performed (X-cite 120LED) at a range of 550–605 nm (43 HE dsRed Zeiss Filter). Images were captured by an ORCA Flash 4.0 LT Monochromatic Digital CMOS camera. Image analysis was completed using MicrobeJ. Fluorescent foci were detected and counted using the maxima feature in MicrobeJ. Maxima are considered the regions of highest fluorescence within a cell. We used this measurement to quantify the amount of oxidized PO1 molecules within cells. Cell length was measured using the medial axis feature in MicrobeJ [24]. At least six fields of view were captured for each strain and condition tested, thus resulting in the imaging of several hundred cells for all conditions.

### 2.7. Statistical Analysis

We used SPSS 24 software for data processing and analysis. To test whether the proportion of stationary-phase revertants that underwent transformation was different from the proportion of non-revertants that underwent transformation, we conducted a chi^2^ test. For the chi-square analysis, we first standardized the number of Spc^R^ colonies for each population to the non-revertant cells that were transformed with DNA (*n* = 1755). Then, we constructed a contingency table to test the independence between the two conditions of transformation (DNA added and No DNA) and the population type (non-revertant, early revertant, and late revertant) and found that these variables were independent (chi-square value 5.04, d.f. 2). Then, we tested the number of transformants in each population type for each condition of transformation separately. In the DNA added condition, we used the number of transformants in the non-revertant population as the expected value; the number of transformants in each of the revertant populations (early and late revertants) was considered the observed value. The chi-square value for the DNA added condition was 208 (d.f. 1), which indicated a significant difference between the non-revertant and revertant populations. The same analysis was repeated in the No DNA added condition and the chi-square value was 3.6 (d.f. 1), which indicated no significant differences among the three populations. The results are presented in percent in Table 3.

For the rest of the experiments, statistical significance was determined by performing ANOVA. Significance was tested at *p* ≤ 0.05. Means were tested using the LSD test at *p* ≤ 0.05. The data from experiments in which cells were untreated or treated with *t*-BHP was LOG transformed to normalize distributions and analyzed by ANOVA. We used a letter system to denote significant differences between means. We assigned “a” to the means that were not significantly different from the mean with the highest value, “b” to means that were different from the “a” group, and so on.

## 3. Results

### 3.1. Stationary-Phase Revertants Are More Likely to Be K-Cells than the Background Population.

To investigate the relationship between the K-state and stationary-phase mutagenesis, we revisited the observations by Sung and Yasbin [2]. They examined the accumulation of mutations in three amino acid biosynthesis genes in stationary-phase cells under nutritional stress over a period of nine days. They conducted these assays in different genetic backgrounds, including *recA* and the general stress sigma factor σ^B^ (σ^S^ in *E. coli*). These genes encode key factors (RecA) or activate genes important in stress-induced mutagenesis in *E. coli* [1].. Those experiments also included the ComA and ComK factors which control the development of competence, a state in which cells incorporate foreign DNA into the chromosome via homologous recombination [2]. Moreover, the inactivation of ComK, which controls the late steps in the formation of the K-state, resulted in a marked decrease of Met^+^ reversions, but the inactivation of ComA, which controls the early steps, did not [2,25]. These observations suggested that the genetic changes producing Met^+^ reversions in stationary-phase *B. subtilis* are promoted by the late steps that differentiate cells into competence in the absence of genetic recombination. Experimentally, we modified our stationary-phase mutagenesis assay to measure the contribution of the development of the K-state among revertants. Briefly, cells of the strain YB955, which is auxotrophic for methionine, were grown to T_90_ (ninety minutes passed the cessation of exponential phase) and prepped for transformation. Then, the plasmid, pDR111, which contains a selectable spectinomycin cassette, was supplied to half of the cells. The spectinomycin cassette is not linked to any of the methionine auxotrophy. The other half of the cells underwent the same preparation but were not exposed to the plasmid. Instead of selecting for the transformation of the plasmid, we plated the cell preparations to select for Met^+^ stationary-phase colonies. We tracked revertant colonies for nine days but considered only those colonies that arose on day 5 or later to be stationary-phase mutants (Additional File 1). Previous experiments that established the system to measure stationary-phase mutagenesis in *B. subtilis* characterized mutants at different times of incubation on selective media and conducted reconstruction experiments. Based on the genetic changes and the growth ability of late revertants in the presence of non-revertants, those reports showed that colonies that arise in the first 3–4 days after the onset of selection are the product of growth-dependent processes [2,26,27]. Then we used day 5 to differentiate revertants as early or late. During this time, the non-revertant background population was measured for its ability to survive (Additional File 1) (see Methods). Those results showed a constant level of CFU throughout nine days.

To determine if the K-state correlated with stationary-phase mutagenesis, both revertant colonies and non-revertant colonies were screened for growth on tryptose blood agar base (TBAB) containing 100 μg/mL spectinomycin, which selects for transformants (Table 3). When we conducted the transformation procedure with the plasmid, we found that 10.9% of early revertants, colonies that arose between days 1 and 4, and 18.1% of late revertants, colonies that arose after day 5, were resistant to spectinomycin. In contrast, only 1.7% of the non-revertant colonies were resistant to spectinomycin. *B. subtilis* cells can become resistant to spectinomycin through a spontaneous single base-pair mutation [28]. To estimate the proportion of colonies that arose through spectinomycin-resistant spontaneous mutations and not through the uptake of the plasmid during the transformation, we patched early revertants, late revertants, and non-revertants that were not exposed to pDR111 during the transformation procedure. We found that when no plasmid DNA was added, 4.0%, 1.0%, and 1.6% of early revertants, late revertants, and non-revertants, respectively, were resistant to spectinomycin (Table 3). The chi-square test results suggest that a majority of the spectinomycin-resistant revertants from the DNA-added condition were due to the development of competence and the uptake of plasmid DNA. These observations are congruent with the finding that the development of the K-state promoted stationary-phase mutagenesis.

### 3.2. Stationary-Phase Mutagenesis Occurs Independently of DNA Uptake

**Figure 1:**
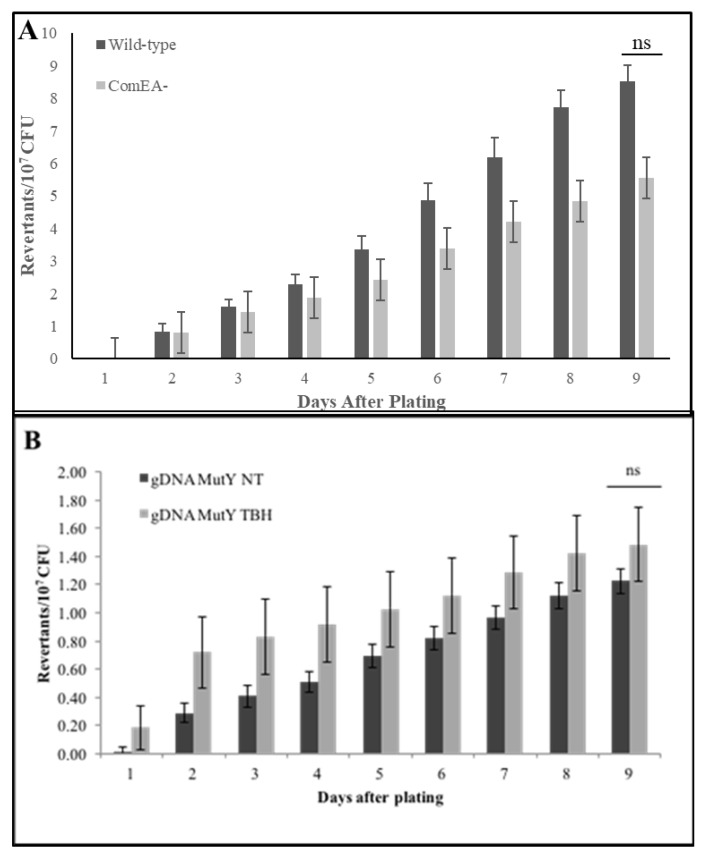
Stationary-phase mutagenesis is DNA-uptake independent. (**A**) The accumulation of stationary-phase Met^+^ revertants under conditions of amino acid starvation in YB955 (parental strain) and JC101 (*comEA^-^*). Student’s *t*-test conducted. (**B**) The accumulation of stationary-phase Met^+^ revertants under conditions of amino acid starvation in YB955 after being supplied with DNA that was not treated (NT) or treated with *tert*-butyl hydroperoxide (TBH) before the DNA was isolated. Data represent the average of at least three separate tests ± standard error of the mean (SEM); ns denotes no significant differences between strains.

To follow up on the previous result (Table 3) and the observations by Sung and Yasbin [2], we tested whether the development of the K-state promoted stationary-phase mutagenesis independently of DNA uptake. We constructed JC101, with a neomycin gene interrupting the *comEA* gene, and measured stationary-phase Met^+^ mutagenesis. The ComEA protein is a component of the competence pore that binds and uptakes DNA during the transformation of *B. subtilis* [29]. The competence pore is a complex of proteins that mediates the binding, translocating, single-stranded forming, and the recombining of foreign DNA into the chromosome. ComEA binds and transports DNA into competent cells during DNA transformation [29]. Cells lacking ComEA are essentially untransformable [29]. We found the ComEA^−^ strain is transformation-defective, as previously reported (Additional File 2). Then, we assayed JC101 and YB955 for their ability to produce stationary-phase revertants and found no significant difference between these two strains (Figure 1A). In addition, there were no differences between the viability of the non-revertant background populations throughout the experiment (Additional File 3). Further, we conducted the stationary-phase mutagenesis assay in the presence of genomic DNA extracted from a YB955 background defective in MutY, a DNA glycosylase that repairs DNA mismatches caused by oxidative damage [30]. Cells lacking MutY have higher mutation rates when exposed to oxidants than parental strains [31]. The DNA donor cells were either treated with *t*-BHP or untreated. The results of those experiments revealed no differences in stationary-phase mutagenesis (Figure 1B). Also, the levels of stationary-phase mutagenesis were like those observed in the absence of DNA. These results agree with previous findings that suggest the development of the K-state, and not recombination nor transformation (nor the transport of oxidized DNA bases), are important in the development of stationary-phase mutants.

### 3.3. Oxidative Stress Potentiates Mutagenesis in K-Cells

The K-state in *B. subtilis* is a bi-stable system, and only ~15% of the cells in a culture develop into this state [32]. To test further whether the K-state promotes stationary-phase mutagenesis, we constructed a YB955 genetic background with an IPTG-inducible *comK* system [16]; this strain was named HAM501. We introduced the *comEA*^-^ mutation into HAM501, which originated AAK502. This system induces all cells in a culture to develop into the K-state when IPTG is supplied exogenously. These strains contain a copy of *comK* under the control of IPTG and express *gfp* under the control of a ComK promoter, which allows for the visual examination of cells undergoing the development of the K-state. We tested the transformation ability of both strains and observed that AAK502 was not transformable even in the presence of damaged DNA (Additional File 4).

To determine if cells lacking ComEA were affected in the expression of the ComK regulon, we grew cultures to an OD_600_ of 0.75 and split them in half. One half was induced with 1 mM IPTG while the other half was not. Cells continued to grow until T_90_, when they were harvested. Induction of the ComK regulon was measured using flow cytometry to assay the amount and intensity of cells expressing GFP under these conditions (Additional File 5). Both HAM501 and AAK502, ComEA^−^, showed a dramatic and equivalent increase in GFP fluorescence in cells induced with IPTG compared to uninduced cells. This result suggests that the interruption of *comEA* does not affect the activity of ComK-regulated genes and rules out the possibility that defects in ComEA have downstream effects on stationary-phase mutagenesis.

**Figure 2:**
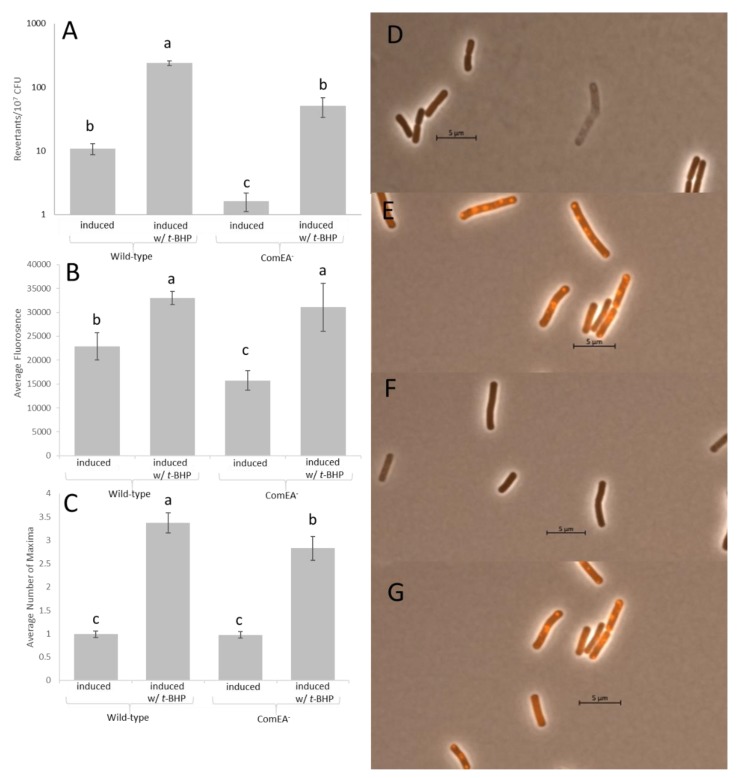
Stationary-phase mutagenesis and fluorescent microscopy with PO1. (**A**) The accumulation of stationary-phase revertants from days 5–9 in K-cells (HAM501) or K-cells lacking ComEA (AAK502) following treatment with either 0 or 1.5 mM *t*-BHP for two hours. (**B**) The average fluorescence within either induced or uninduced wild-type cells (HAM501) or cells lacking ComEA (AAK502) following treatment with either 0 or 5 mM *t*-BHP for two hours. (**C**) The average number of PO1 maxima within either induced or uninduced wild-type cells (HAM501) or cells lacking ComEA (AAK502) following treatment with either 0 or 5 mM *t*-BHP for two hours. (**D**) A representative image of induced wild-type cells (HAM501) following 0 mM *t*-BHP for two hours. (**E**) A representative image of induced wild-type cells (HAM501) following 5 mM *t*-BHP for two hours. (**F**) A representative image of induced cells lacking ComEA (AAK502) following 0 mM *t*-BHP for two hours. (**G**) A representative image of induced cells lacking ComEA (AAK502) following 5 mM *t*-BHP for two hours. Lower case letters were used to denote significant differences between means. “a”, “b”, and “c” are significantly different mean groups.

Recent work has demonstrated the importance of reactive oxygen species (ROS) in the generation of stationary-phase mutations [31]. Interestingly, PerR, a transcription factor activated in response to increases in ROS stabilizes ComK levels in the cell [33]. This prompted us to investigate the roles of ROS in combination with the development of the K-state in the formation of stationary-phase revertants. Therefore, we modified our traditional stationary-phase mutagenesis assay to include exposure to the oxidant, *t*-BHP, and transcriptional induction of the *comK* gene. Briefly, HAM501 and AAK502 (ComEA^−^) cells were grown to T_90_, induced into the K-state by supplying IPTG, exposed to 0- or 1.5-mM *t*-BHP for two hours, washed twice, and plated on minimal medium lacking methionine. The results over the nine days are presented in Additional File 6. Figure 2A presents the number of revertants from days 5 thru 9, which shows, as expected, that the number of revertants was increased when both strains were exposed to *t*-BHP. However, in the absence of *t*-BHP treatment, cells lacking ComEA accumulated fewer mutants than ComEA^+^ cells. Strikingly, when K-cells were damaged with *t*-BHP, ComEA^+^ cells accumulated significantly more mutations than those that lack ComEA (Figure 2A). The background non-revertant population was measured for its viability (Additional File 6), and those data suggest that long-term survival of the strains in selective media is unaffected between strains. Furthermore, the ability of AAK502 (ComEA^−^) to revert to the revertant levels of HAM501 (ComEA^+^) in conditions in which cells were not treated with *t*-BHP (Figure 2A) indicates that stationary-phase mutagenesis is independent of DNA uptake. These results suggest that K-cells can engage in a pro-mutagenic process triggered by oxidative damage and that the presence of a functional DNA uptake system increases oxidative damage and the pro-mutagenic process.

**Figure 3:**
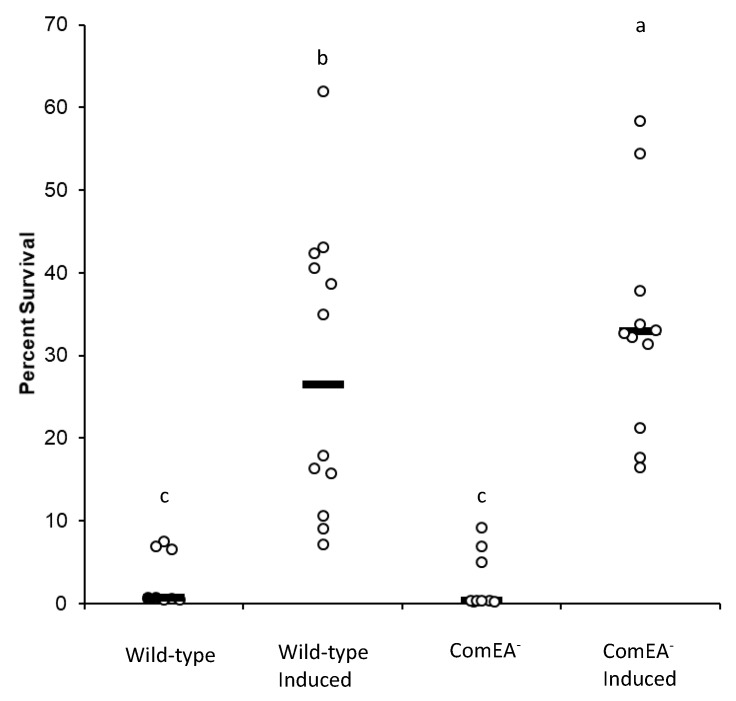
Cell survival following two-hour exposure to 5 mM *t*-BHP. Percent cell survival in the wild-type strain (HAM501) and ComEA^−^(AAK502) strains either treated with 0 or 1 mM IPTG following treatment with 5 mM *t*-BHP. At least nine replicates were completed for each condition. Lower case letters were used to denote significant differences between means. “a”, “b”, and “c” are significantly different mean groups.

To test whether the observed increased stationary-phase mutagenesis levels in K-cells are associated with increased tolerance to and repair of cellular damage caused by ROS, we conducted cell survival and microscopy assays in untreated cells or cells exposed to *t*-BHP and in conditions in which cultures were non-induced or induced into the K-state (addition of IPTG). Survival assays indicated that tolerance to oxidative damage was increased in K-cells compared to non-K-cells (Figure 3). Within the K-cell population, ComEA^−^ cells survived oxidant exposure better than ComEA^+^ cells, with the former cells surviving at an average of 36.4% and the later cells surviving at an average of 28.2%. Interestingly, when treated with *t*-BHP, ComEA^−^ cells showed significantly fewer maxima than their ComEA^+^ counterparts (Figure 2C). Even so, ComEA^−^ cells showed less fluorescence than ComEA^+^ cells, albeit not significantly (Figure 2B). Altogether, these results suggest that the development of the K-state, which includes modifying the cell surface for the uptake of DNA, activates a mechanism that protects against the noxious effects inflicted by oxidants, which includes DNA lesions. One associated consequence of the activation of this mechanism is an increase in mutagenesis in cells under selection.

## 4. Discussion

Previous work has demonstrated that ComK is important for the accumulation of mutations during stress conditions in *B. subtilis* [2]. However, unlike in *Escherichia coli*, this process is independent of recombination [1,2]. Historically, the development of competence was synonymous with genetic transformation. Yet, findings by Berka et al. showed that the K-state was more dynamic than simply a precursor to transformation [15]. These disparate observations prompted us to investigate how the K-state promotes stationary-phase mutagenesis in the absence of genetic recombination [2]. Stationary-phase mutagenesis mechanisms provide insights into evolution and can explain how bacterial cells develop genetic adaptation to escape from host defenses and antibiotic treatment.

First, our experiments showed that the population of stationary-phase revertants had a higher proportion of transforming DNA than the non-revertants from experiments that examined mutations in different genes (Table 3, Additional File 1). We interpreted this result to indicate that the changes associated with the development of the K-state were conducive to stationary-phase mutagenesis. Gene expression and genetic studies demonstrated that the competence state activates genes that respond to DNA damage, most notably the SOS system, DNA uptake and recombination pathways, and oxidative stress [34,35]. Previous experiments showed that the RecA protein was not involved in stationary-phase mutagenesis, which led us to further study how the K-pathway influences stationary-phase mutagenesis.

Next, we tested the possibility that the uptake of exogenous DNA, another trademark characteristic of the K-state, influences stationary-phase mutagenesis. However, the DNA-uptake defective strain showed no significant differences in stationary-phase mutagenesis levels when compared to its wild-type counterpart. This result suggested that the process of DNA binding and uptake are dispensable to the formation of stationary-phase revertants (Figure 1). Interestingly, we noted that the non-transformable ComEA^−^ strain accumulated slightly less revertants than the wild type. Perhaps, the modification of the cell surface (assembly of a functional DNA uptake system) predisposes the cell to cytotoxic and genotoxic damage, which in turn activates a protective, error-prone DNA repair response that produces mutations. Interestingly, studies that examine protein networks and endogenous DNA damage revealed that increases in reactive oxygen species are associated with the presence of transmembrane transporters in *E. coli* [36]. Further, reports examining K-cells in *B. subtilis* showed activation of detoxication mechanisms [15,34].

To test the idea that the presence of a functional DNA uptake system increases oxidative damage in cells and consequently stationary-phase mutagenesis, we tested cultures induced to develop into the K-state for their ability to generate stationary-phase mutants in the absence and presence of ComEA, and as affected by exposure to the oxidant *tert*-butyl hydroperoxide. The results indicated the following. (1) In the absence of ComEA and t-BHP treatment, K-cells still produced stationary-phase mutants. (2) ComEA^+^ cells accumulated more mutants than cells lacking this protein. (3) When cells were exposed to an oxidant, the same response pattern was observed in stationary-phase mutagenesis (Figure 2A). Also, we quantified cell survival and cell damage via florescence after exposure to *t*-BHP. Our results showed that induction of the K-state resulted in increased cell survival to oxidant exposure (Figure 3). One likely explanation for this outcome is that K-cells activate genes that detoxify compounds [15]. However, one interesting finding was that ComEA^+^ cells displayed a lower cell survival than ComEA^−^ cells, which suggested that the presence of a functional DNA uptake system predisposes cells to oxidative damage.

We visualized cells after exposure to *t*-BHP by using a fluorescent probe that responds to oxidative damage. The average maxima were significantly higher in ComEA^+^ cells than in ComEA^−^ cells after exposure to *t*-BHP, (Figure 2C). These results suggest that producing a functional DNA uptake apparatus compromises the cell’s ability to withstand oxidative damage. Altogether, we conclude that the activation of the K-state leads to the alteration of the cell surface and that such changes predispose the cell to accumulate oxidative damage, as measured by fluorescent maxima in *t*-BHP treated cells (Figure 2C). This notion is supported by reports that showed overlap between the regulons activated in the K-cell state and cells experiencing oxidative or cell envelope stress [15,37,38]. The increase in oxidative damage is followed by activation of mechanisms that actively repair DNA lesions [15]. This assertion is supported by the increased survival to the oxidant observed in the K population of cells (Figure 3). We speculate that the DNA repair reactions activated in K-cells occur via error-prone repair as attested by the increased stationary-phase mutagenesis levels in such cells (Figure 2A). This stationary-phase mutagenic process is independent of the process of genetic transformation, which suggests that the K-state increases genetic diversity via Rec-dependent and Rec-independent pathways.

The observations presented here, in combination with research that showed that highly transcribed genes accumulate mutations in cells under oxidative stress [31], supports a concept in which stressed *B. subtilis* cells limit increased mutagenesis to a fraction of cells and to genomic regions under elevated transcriptional activity. This strategy increases the potential to gain fitness and minimizes risks in *B. subtilis*, quintessentially known to adapt to stress via the development of different subpopulations [39]. *E. coli* employs a different strategy to mitigate the risks associated with increases of genetic diversity; mutagenic break repair happens in regions suffering double-stranded breaks [40]. Spontaneous double-strand breaks happen in a fraction of the cell population and the likelihood of it occurring is increased in transcribed regions [5,9]. This is supported by reports that demonstrated factors influencing transcription termination affected stress-induced mutagenesis in *E. coli* [10,41]. Interestingly, oxidative damage to DNA has been implicated in stress-induced and stationary-phase mutagenesis in both bacterial models, but the pathways that process this damage and produce mutations are not evolutionarily conserved. Therefore, minimizing the risks associated with increased mutagenesis in stressed cells exemplifies convergent evolution [7]. These findings support a scenario in which cells can regulate the ability to evolve (reviewed in [7]) and provide insights in the formation of mutations that, in bacterial pathogens, can lead to the formation of antibiotic resistance [42] and increased fitness that prolong chronic infections [43].

## Figures and Tables

**Table 1:** Strains and plasmids used in this study.

Strain Name	Genotype	Reference or Source
***Bacillus subtilis* strains**		
YB955	*hisC952 metB5 leuC427* xin-1 Spβ ^SENS^	[2]
JC101	YB955 *comEA::neo*	This study
HAM501	*YB955 amyE::P_hs_—comK (sp) comK-gfp (CBL, cm)*	This study
HAM502	HAM501 *hom::erm*	This study
AAK502	HAM501 *comEA::neo*	This study
BD4010	*his leu met amyE:: P_hs_–comK (spc*) *comK-gfp (cat)*	[16]
***Escherichia coli* strain**		
Mon1	*endA1 recA1 mcrA ∆(mrr-hsdRMS-mcrBC) ϕ80lacZ∆M15∆lacX74 ∆(ara,leu7697 ara ∆139 galU galK nupG rpsL F- λ-*	Monserate Biotechnology Group (San Diego, CA)
**Plasmids**		
pDR111	Integrative plasmid, confers Sp^R^	David Rudner
pBEST502	Integrative plasmid, confers Nm^R^	[17]
pDG1664	Integrative plasmid, confers MLS^R^	[18]

**Table 2:** Primers used in this study.

Primer Name	Primer Sequence
*comEA* A fwd	agctggaagctttaaggtaacgctcttgccag
*comEA* A rvs	agccgtgtcgacaaatttacttgcgcttcgtc
*comEA* B fwd	agtcacggatcctctgcaggacgggacagtgg
*comEA* B rvs	aagcctcgagctctccatcagtcggcaccccaaac

^1^ Underlined sequences correspond to restriction sites.

**Table 3:** Percentage of stationary-phase revertants that were resistant to spectinomycin.

Condition	Early Revertants (From Days 1–4)	Late Revertants (From Days 5–9)	Non-Revertants
DNA added	10.9% * n = 550	18.1% * n = 545	1.7% *n* = 1755
No DNA added	4.0% *n* = 100	1.0% *n* = 100	1.6% *n* = 440

* Denotes chi-square test of *p* < 0.05. See the statistical analysis section for more details.

## Supplementary Materials

The following are available online at https://www.mdpi.com/2073-4425/11/2/190/s1, Additional File 1: The accumulation of stationary-phase mutations under conditions of amino acid starvation in YB955 (parental strain) after addition of a transformation marker pDR111. Additional File 2: Number of colonies resistant to spectinomycin following transformation with pDR111. The top table shows results for the wild-type cells (YB955). The bottom table shows results for the cells lacking ComEA (JC101). Additional File 3: Non-revertant background viability from Figure 1. Additional File 4: Number of colonies resistant to erythromycin following transformation into HAM501 (ComEA+) or AAK502 (ComEA-) with DNA from HAM502 (HAM501 hom::erm) cells that were treated with 10 mM of *t*-BHP for two hours before the DNA was isolated. Additional File 5: The fluorescence intensity of cells following induction measured by flow cytometry. The top panel of A shows the uninduced wild-type cells (HAM501), whereas the bottom panel of A shows the uninduced cells lacking ComEA (AAK502). The top panel of B shows the induced wild-type cells (HAM501), whereas the bottom panel of B shows the induced cells lacking ComEA (AAK502). Additional File 6: (A) Accumulation of Met+ revertants over nine days in the wild-type (HAM501) and the ComEA- mutant (AAK502); all conditions were induced with IPTG (i) and half were exposed to 1.5 mM *t*-BHP for two hours; (B) Viability of the wild-type (HAM501) and ComEA-deficient (AAK502) cells either treated with 0 or 1.5 mM *t*-BHP over the nine-day stationary-phase mutagenesis assay.
