# Supplementary material for: The Bacillus Subtilis K-State Promotes Stationary-Phase Mutagenesis via Oxidative Damage
Source: Genes (Basel). 2020 Feb 11;11(2):190. doi: 10.3390/genes11020190 (PMC7073564; doi:10.3390/genes11020190)
Supplement: Supplementary file 1 [file genes-11-00190-s001.zip › Additional File 1 HM.docx]

A

B

Additional File 1. The accumulation of stationary-phase mutations under conditions of amino acid starvation in YB955 (parental strain) after addition of a transformation marker pDR111. (A) The trend of the accumulation of revertants is constant with previous SPM experiments. (B) The non-revertant background remains constant throughout the 9 days. Data represents the average of three separate tests ± standard error of the mean (SEM).
