# Supplementary material for: The Bacillus Subtilis K-State Promotes Stationary-Phase Mutagenesis via Oxidative Damage
Source: Genes (Basel). 2020 Feb 11;11(2):190. doi: 10.3390/genes11020190 (PMC7073564; doi:10.3390/genes11020190)
Supplement: Supplementary file 1 [file genes-11-00190-s001.zip › Additional File 2.docx]

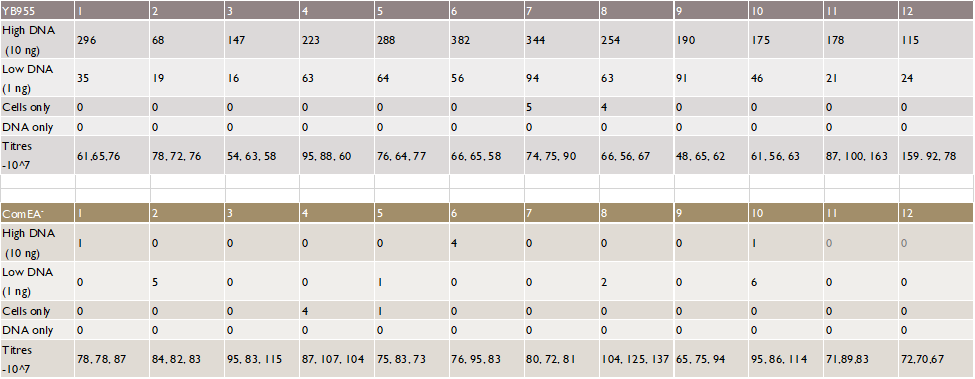


Additional File 2. Number of colonies resistant to spectinomycin following transformation with pDR111. The top table shows results for the wild-type cells (YB955). The bottom table shows results for the cells lacking ComEA (JC101).
