# Supplementary material for: The Bacillus Subtilis K-State Promotes Stationary-Phase Mutagenesis via Oxidative Damage
Source: Genes (Basel). 2020 Feb 11;11(2):190. doi: 10.3390/genes11020190 (PMC7073564; doi:10.3390/genes11020190)
Supplement: Supplementary file 1 [file genes-11-00190-s001.zip › Additional File 3.docx]

A


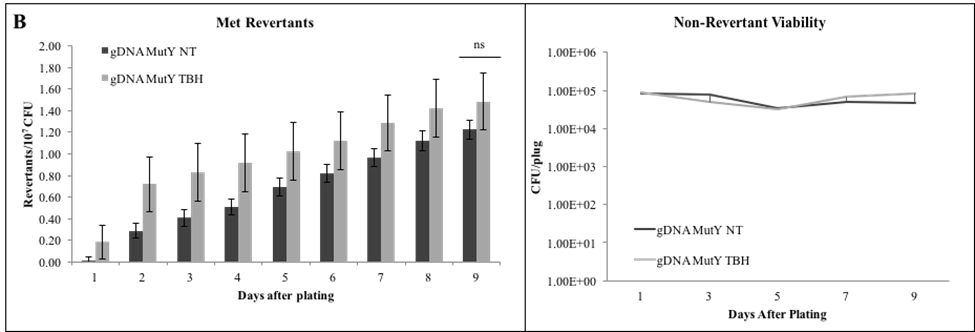


B

Additional File 3. Non-revertant background viability from Figure 1. A) Viability of the wild-type (YB955) and ComEA-deficient cells over the nine-day stationary-phase mutagenesis assay. B) Viability of YB955 cells supplied with either DNA from a MutY-deficient strain treated with either 0- or 1.5-mM *t*-BHP for two hours before the DNA was isolated. Each point represents an average of five samples.
