# Supplementary material for: The Bacillus Subtilis K-State Promotes Stationary-Phase Mutagenesis via Oxidative Damage
Source: Genes (Basel). 2020 Feb 11;11(2):190. doi: 10.3390/genes11020190 (PMC7073564; doi:10.3390/genes11020190)
Supplement: Supplementary file 1 [file genes-11-00190-s001.zip › Additional File 4.docx]

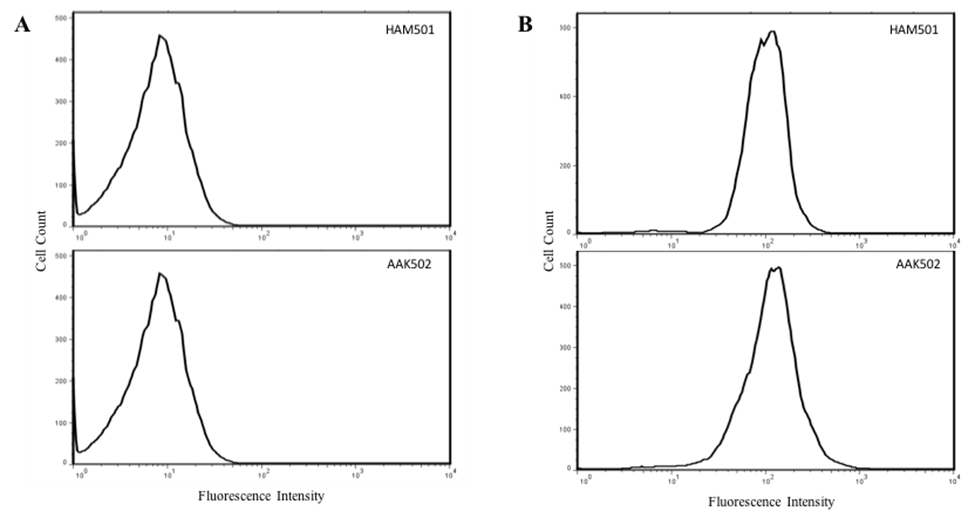


Additional File 4. The fluorescence intensity of cells following induction measured by flow cytometry. The top panel of A shows the uninduced wild-type cells (HAM501), whereas the bottom panel of A shows the uninduced cells lacking ComEA (AAK502). The top panel of B shows the induced wild-type cells (HAM501), whereas the bottom panel of B shows the induced cells lacking ComEA (AAK502).
