# Supplementary material for: The Bacillus Subtilis K-State Promotes Stationary-Phase Mutagenesis via Oxidative Damage
Source: Genes (Basel). 2020 Feb 11;11(2):190. doi: 10.3390/genes11020190 (PMC7073564; doi:10.3390/genes11020190)
Supplement: Supplementary file 1 [file genes-11-00190-s001.zip › Additional File 5 HM new.docx]

A

B

Additional File 5. A) Accumulation of Met^+^ revertants over nine days in the wild-type (HAM501) and the ComEA^-^ mutant (AAK502). All conditions were induced with IPTG (i) and half were exposed to 1.5 mM *t*-BHP for two hours. B) Viability of the wild-type (HAM501) and ComEA-deficient (AAK502) cells either treated with 0 or 1.5 mM *t*-BHP over the nine-day stationary-phase mutagenesis assay.
