# Supplementary material for: The Bacillus Subtilis K-State Promotes Stationary-Phase Mutagenesis via Oxidative Damage
Source: Genes (Basel). 2020 Feb 11;11(2):190. doi: 10.3390/genes11020190 (PMC7073564; doi:10.3390/genes11020190)
Supplement: Supplementary file 1 [file genes-11-00190-s001.zip › Additional File 6..docx]

| **Trial** | **Titres (10^-7^)** | **Transformants** | **Cells Only** | **DNA Only** |
| --- | --- | --- | --- | --- |
| AAK502 - A | 51, 51 | 0, 0 | 0 | 0 |
| AAK502 - B | 35, 38 | 0, 0 | 0 | 0 |
| AAK502 - C | 49, 61 | 0, 0 | 0 | 0 |
| AAK502 - D | 34, 60 | 0, 0 | 0 | 0 |
| HAM501 - A | 50, 73 | 31, 21 | 0 | 0 |
| HAM501 - B | 94, 127 | 1, 1 | 0 | 0 |
| HAM501 - C | 81, 60 | 10, 14 | 1 | 0 |
| HAM501 - D | 51, 41 | 20, 26 | 4 | 0 |

Additional File 6. Number of colonies resistant to erythromycin following transformation into HAM501 (ComEA^+^) or AAK502 (ComEA^-^) with DNA from HAM502 (HAM501 *hom::erm*) cells that were treated with 10 mM of *t*-BHP for two hours before the DNA was isolated.
